# Thymoquinone protects against cardiac mitochondrial DNA loss, oxidative stress, inflammation and apoptosis in isoproterenol-induced myocardial infarction in rats

**DOI:** 10.1016/j.heliyon.2021.e07561

**Published:** 2021-07-14

**Authors:** Asmaa A. Khalifa, Radwa M. Rashad, Wessam F. El-Hadidy

**Affiliations:** a Department of Pharmacology and Therapeutics, Faculty of Pharmacy, Pharos University in Alexandria, Egypt; b Department of Pathology, Medical Research Institute, Alexandria University, Alexandria, Egypt; c Department of Pharmacology and Experimental Therapeutics, Medical Research Institute, Alexandria University, Alexandria, Egypt

**Keywords:** Isoproterenol, Thymoquinone, Cardiopotection, Cardiac mtDNA, Oxidative stress, Inflammatory cytokines, Apoptotic markers, Masson trichrome staining

## Abstract

**Introduction:**

Myocardial infarction (MI) is an ischemic life-threatening disease with exaggerated oxidative stress state that vigorously damages the cardiomyocyte membrane and subcellular structures, including the vital mitochondrial DNA (mtDNA). The mtDNA is responsible for the proper functionality of the mitochondria, which are abundant in cardiomyocytes due to their dynamic nature and energy production requirements. Furthermore, oxidative stress triggers an inflammatory cascade and eventual apoptosis, which exacerbates cardiac injuries and dysfunction.

**Aim:**

The present study used an isoproterenol (ISP)-induced MI rat model to investigate the role of the main active constituent of Nigella Sativa seeds, thymoquinone (TQ), in preserving the cardiac mtDNA content and ameliorating oxidative stress, inflammation, and apoptosis.

**Methods:**

Rats in the (TQ + ISP) group were pre-treated with TQ (20 mg/kg/day) for 21 days before the MI induction using ISP (85 mg/kg/day). In addition, negative control and ISP groups were included in the study for comparison. A histopathological examination was performed and serum cardiac parameters (cTnI and LDH) were assessed. In addition, mtDNA content, oxidative stress parameters (MDA, GSH, SOD, GPx, and CAT), inflammatory mediators (IL-6, IL-1β, and TNF-α), and apoptosis markers (BAX, Bcl2, and caspase-3) were detected.

**Results:**

The results showed that pre- and co-treatment with TQ in the (TQ + ISP) group reversed the histoarchitecture changes, caused a significant decrease in serum cardiac markers, oxidative stress markers, inflammatory cytokines, the apoptosis process, and preserved the cardiac mtDNA content.

**Conclusion:**

TQ is a cardioprotective agent with an extended effect on preserving the cardiac mtDNA content, in addition to its powerful antioxidant, anti-inflammatory, and anti-apoptotic action.

## 1 Introduction

Myocardial infarction (MI) is one of the leading causes of morbidity and mortality worldwide [1], which occurs due to coronary artery occlusion and an imbalance between cardiac demand and cardiac supply [2]. The heart has a limited capacity for anaerobic metabolism and cannot cope with blood, nutrients, and oxygen deprivation during MI attack [3], which results in pathological changes leading to eventual cardiac dysfunction [4].

A high amount of free radicals are produced following the shortage of cardiac supply [5], which react with the membrane lipids, proteins and break DNAs exerting an oxidative stress state [6, 7]. Cardiomyocyte injury occurs when oxidative stress is beyond the capacity of the internal defensive enzymatic (superoxide dismutase SOD, catalase CAT and glutathione peroxidase GPx) and non-enzymatic (reduced glutathione GSH) antioxidants [8].

The inflammatory response in MI includes increased expression of pro-inflammatory cytokines such as tumor necrosis factor-α (TNF-α), which causes an increase in cytokines, chemokines, and adhesion molecules. It also causes gene upregulation of Interleukin 1-β (IL-1β), the gatekeeper of the process of inflammation [9] together with interleukin-6 (IL-6) which decreases the basal contractility of the myocytes accompanied by the affection of beta-adrenergic receptor responsiveness [10].

Cardiomyocytes contain a high number of mitochondria to help in coping with increased energy requirements by these dynamic cells, while the dysfunction of mitochondria will negatively affect cardiac functionality [11]. Mitochondria are known to have their own circular DNA [12] which is highly labile for injury by the excessive release of ROS. This high susceptibility of the circular mitochondrial DNA (mtDNA) is due to being close to the inner mitochondrial membrane and the lack of repair pathways in such a type of DNA [13]. Moreover, mitochondrial DNA (mtDNA) damage results in a continuous cycle of additional ROS production which exaggerates oxidative stress damage and further mitochondrial dysfunction [14]. In addition, portions of mtDNA are released into the circulation and act as pro-inflammatory substances which exaggerate the inflammatory injury [15, 16].

Apoptosis, the programmed cell death, is the type of cell death that is mediated by extrinsic as well as intrinsic pathways following oxidative stress and inflammatory damage [17]. The intrinsic pathway triggers apoptosis in response to apoptotic signals, pro-apoptotic proteins such as Bax and Bak, and anti-apoptotic proteins such as Bcl-2 which alter the membrane permeability of the mitochondria leading to changes in membrane potential, cytochrome *c* release, and caspase-3 formation with excessive production of free radicals [18, 19].

Recently, herbal medicine use has expanded worldwide for many diseases [20]. A herb that has potential cardioprotective effects is *Nigella Sativa (NS), family Ranunculaceae* seeds [21, 22]. The main active constituent in *NS* is thymoquinone (TQ) [23], which is responsible for the antioxidant and anti-inflammatory properties of NS [24]. Our study was designed to investigate the role of TQ in ameliorating oxidative stress, inflammation, and apoptosis as well as the protective effect on cardiac mtDNA in experimentally induced (MI).

## 2 Material and methods

### 2.1 Animals

Twenty-four adult male albino rats weighing 200–250 g were used in this study. They were housed on wood shavings in metal cages for animals, four per cage, with free access to water and food. Animals were kept under the same standard environmental conditions of light and temperature and were cared for. The experimental design of the present study was approved by the Animal Care and Use Committee, Medical Research Institute, Alexandria University. The procedures, dosing, and handling related to the animals in the study were carried out according to the guidelines of the international council for laboratory animal science (ICLAS) and International Association of Colleges of Laboratory Animal Medicine (IACLAM) [25].

**Chemicals:** Thymoquinone (TQ): Thymoquinone powder (Sigma- Aldrich, Germany). Isoproterenol (ISP): Isoproterenol powder (Sigma- Aldrich, Germany). Dimethyl Sulfoxide DMSO prepared as 5% solution, (Arabic laboratory Equipment Co. Cairo, Egypt).

### 2.2 Experimental design

#### 2.2.1 Rats were randomly assigned into 3 groups (8 rats/group)

**Group I (Control group):** the rats of this group received 1 ml/day of 5% DMSO orally for 21 days. In addition, they received 0.5 ml/day of normal saline, subcutaneously on the 20^th^ and 21^st^ days, at a 24 hours interval.

**Group II (Isoproterenol group, ISP group**): the rats of this group received 1 ml/day of 5% DMSO via oral gavage for 21 days. MI was induced by dissolving isoproterenol ISP (85 mg/kg/day) in normal saline and injecting it subcutaneously, at 24 hour intervals on the last two days of the experiment [26, 27].

**Group III (Thymoquinone and Isoproterenol group, ISP + TQ20 group**): the rats of this group were treated with 20 mg/kg/day of Thymoquinone (TQ) via oral gavage for 21 days [27]. Then, on the 20^th^ and 21^st^ days, they were given ISP for the induction of MI by the same dose and route of administration mentioned in group II.

#### 2.2.2 Serum and homogenate parameters

All the animals survived until the end of the experiment. Twenty-four hours after the second subcutaneous injection of ISP (or normal saline), six rats were randomly selected from each group and anesthetized using thiopental sodium anesthesia (30 mg/kg, i.p.). Animals were sacrificed and the blood was collected from the abdominal aortae and the heart tissues were excised. Blood samples were left to coagulate at 37 °C for 1 hour until clot retraction occurred. Serum samples were separated by centrifugation at 3000 r.p.m for 15 min, divided into aliquots, and then used for the determination of serum parameters. The heart tissue excised from each rat was homogenized in phosphate-buffered saline (PBS, pH 7.4) to prepare 10% homogenates divided into aliquots and stored at -80 °C until used for assay of the biochemical parameters in homogenates.

#### 2.2.3 Assay of serum cardiac markers, cardiac troponin (cTnI) and lactate dehydrogenase (LDH) levels

The cardiac troponin I (cTnI) level and lactate dehydrogenase (LDH) level were estimated spectrophotometrically at 450 nm using commercially available enzyme-linked immunosorbent assay (ELISA) kits. Rat Cardiac Troponin I was determined using a competitive ELISA kit, Catalog No.: MBS727624 (My Biosource Co, USA) [28], and LDH was determined using a quantitative sandwich ELISA kit, Catalog No.: MBS043166 (My Biosource Co, USA) [29] was used respectively.

#### 2.2.4 Histopathological examination

Two rats from the total of eight rats per group were randomly selected, anesthetized, and sacrificed by the same method and at the same time mentioned in the experimental design and assigned specifically for the histopathological examination. The hearts of the two rats were excised and immediately washed with ice-cold saline. The hearts were fixed with 10% neutral buffered formalin, and then embedded in paraffin. Sections of 4 μm thickness were stained with hematoxylin and eosin (H&E stain) and Masson trichrome [30, 31].

#### 2.2.5 Assay of tissue oxidative stress markers by colorimetric technique

The extent of lipid peroxidation was assayed by the thiobarbituric acid method described by Ohkawa et al. [32], based on the reaction between malondialdehyde (MDA), the most abundant aldehyde product of lipid peroxidation, and thiobarbituric acid. The content of the reduced glutathione (GSH) in cardiac tissue homogenate was determined spectrophotometrically using the method described by Richardson and Murphy, which is based on the reductive cleavage of Ellman's reagent (5,5′-dithiobis-2-nitrobenzoic acid) by the SH group of glutathione to yield a yellow color [33]. While superoxide dismutase (SOD) activity was detected by Nishikimi et al., a method [34] based on the ability of the enzyme to inhibit phenazine methosulphatemediated reduction of nitroblue tetrazolium dye. Glutathione peroxidase (GPx) activity was determined by the method described by Flohe and Gunzler, which is based on monitoring the generation of GSH from GSSG by the action of glutathione reductase in presence of NADPH [35]. The catalase activity was detected using the Luck method that is based on the disappearance of hydrogen peroxide by the action of catalase concerning time [36].

#### 2.2.6 Assay of inflammatory mediators: interleukin-1 β (IL-1β), interleukin-6 (IL-6) and tumor necrosis factor-alpha (TNF-α) by enzyme-linked immunosorbent (ELISA) technique

Interleukin 1β, IL-6 and TNF-α in heart homogenate were determined using quantitative sandwich ELISA kit Cat No. MBS825017 (My Biosource Co, USA) for ILl-β [37] Catalog No.: MBS355410 for IL-6 (My Biosource Co, USA) and Catalog No.: MBS355371 for TNF-α (My Biosource Co, USA) [38, 39]. Samples were estimated spectrophotometrically at 450 nm.

#### 2.2.7 Assay of apoptosis marker (Bax) by western blotting technique and (Bcl-2) and caspase-3 using enzyme-linked immunosorbent (ELISA) technique

Bax protein was extracted from the homogenate samples using RIBA lysis buffer PL005 then quantitative protein analysis was determined by the Bradford Protein Assay Kit (SK3041) buffer and the kit was provided by Bio BASIC INC. (Marhham Ontario L3R 8T4 Canada). The TGX Stain-Free™ FastCast™ Acrylamide Kit (SDS-PAGE) which was provided by Bio-Rad Laboratories, TNC, USA Catalog. NO.161-0181 was used in our study for the preparation of SDS-PAGE (Sodium Dodecyl Sulfate Polyacrylamide Gel ELectrophoresis). From the total extracted protein, 20 μg was loaded per mini-gel well. A molecular weight marker (BLUelf pre-stained protein ladder, GeneDireX, Taiwan, Cat No.PM008-0500) was used to enable the determination of the protein size and also to monitor the progress of an electrophoresis run. Protein bands were transferred from gel to membrane using the Bio-Rad Trans-Blot Turbo. Immediately, the blot separation was visualized and imaged using stain-free blot technology and the ChemiDoc TM imager. The membrane was blocked by tris-buffered saline with Tween 20 buffer and 3% bovine serum albumin (BSA). The blot was incubated with anti-Bax (1:2000, Thermofisher USA) as a primary antibody, and then incubation was done with the HRP-conjugated as a secondary antibody (Goat antirabbitIgG- HRP-lmg Goat mab-Novus Biologicals) solution against the blotted target protein. Image analysis software was used to read the band intensity of the target proteins against a control sample of β actin (1:2000, Thermofisher, USA) on the ChemiDoc MP imager [40]. The concentration of (Bcl-2) in cardiac tissue homogenates was estimated spectrophotometrically using a rat quantitative sandwich ELISA kit with catalog number CSB-E08854r (Cusbio Co., China). Also, the caspase-3 concentration in heart homogenates was determined using the rat caspase-3 ELISA Kit. Cat No. MBS261814 (My Biosource Co, USA) [41].

#### 2.2.8 Assay of mitochondrial DNA (mtDNA) concentration using a conventional PCR technique

Extraction of DNA was done using a Qia-amplification DNA extraction kit supplied by (Qiagen, USA) according to the manufacturer's instructions. The extracted DNA concentration was read at 260 nm on UV-spectrophotometer supplied by (Beckman, USA). Amplification of the mitochondrial DNA by PCR with the appropriate primers for:

***mtDNA Forward primer: 5-CACTTTCCACACAGACATCA-3*.**

***mtDNA Reverse primer: 5-TGGTTAGGCTGGTGTTAGGG-3*.**

The isolated DNA from each sample was used for amplification. The reaction mixture was prepared by adding 12.5 μl of DreamTaqPCR Master Mix, 0.5 μl of forward and reverse primer (10 μM), and 10.5 μl RNase-freeH_2_O. Also, 2 μl of DNA template was added. Using a Thermal Cycler (such as LifePro Thermal Cycler, Bioer), PCR was performed with an initial denaturation at 94 °C for 15 min (1 cycle), followed by denaturation at 94 °C for 30 s, annealing at 60 °C for 30 s, and extension to 72 °C for 1 min and 30s (30 cycles). The final extension was performed at 72 °C for 7 min and the concentration of mtDNA was determined in ng/μl [42].

### 2.3 Statistical analysis

All values in figures and tables are expressed as mean ± standard error of the mean (SEM). The computer package SPSS 11.5 (SPSS Inc, Chicago, IL, USA) was used for data analysis. The analysis of variance (one-way ANOVA classification) method was used for comparing the experimental groups, followed by Tukey's multiple comparisons between the three experimental groups. The correlation between variables was tested by computing the correlation coefficient (r, Pearson's test). The significance of the difference was set at *p* < 0.05.

## 3 Results

### 3.1 Effect of TQ pretreatment on serum cardiac biomarkers

**Figure 1:**
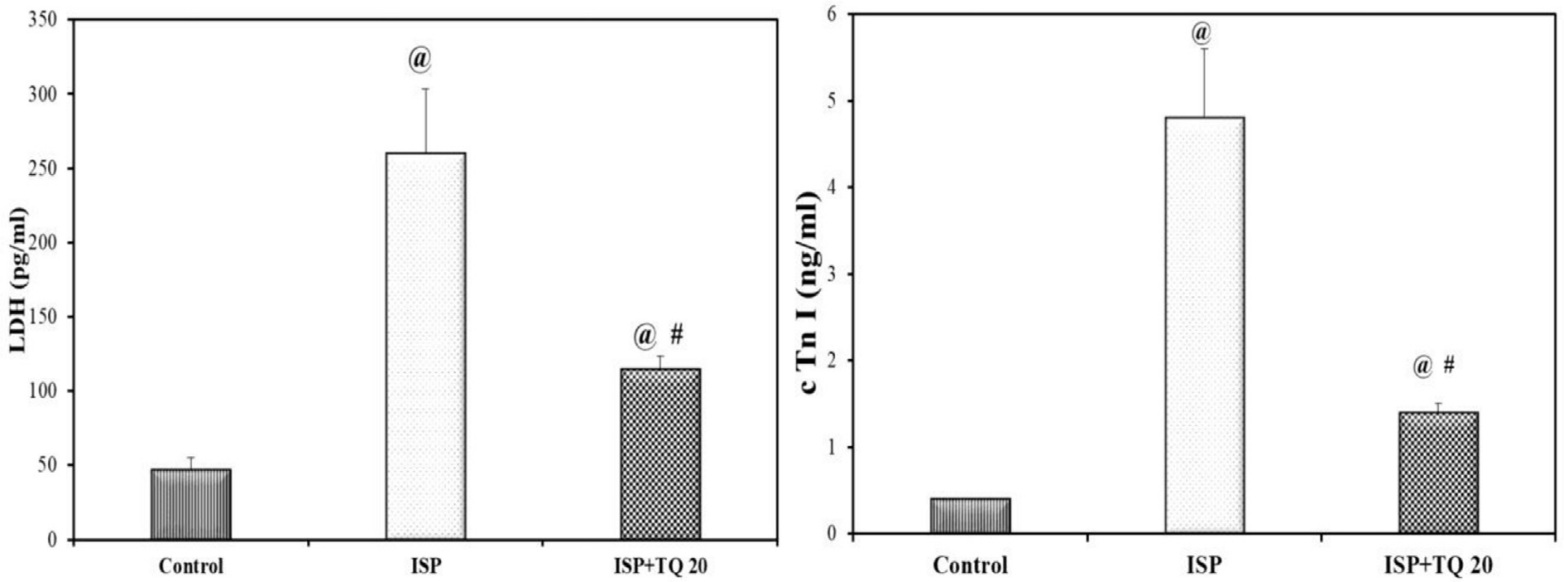
Effect of thymoquinone use on lactate dehydrogenase; (LDH), and troponin I; (cTn I) as serum biomarkers of myocardial infarction. Values are Mean ± SEM, n = 6; @: compared with control, p < 0.05; #: with ISP group, p < 0.05.

The isoproterenol-treated group showed significant elevation in both serum LDH lactate dehydrogenase and cardiac troponin I (cTnI) levels as compared to normal control rats. In contrast, rats pre-and co-treated with TQ 20 mg/kg for 21 days (ISP + TQ20 group) showed a significant decline in the serum levels of the LDH and (cTnI) when compared to the ISP-treated group (F = 139.37 and F = 198.78, p < 0.001 respectively), Figure 1.

### 3.2 Effects of TQ pretreatment on histopathology

**Figure 2:**
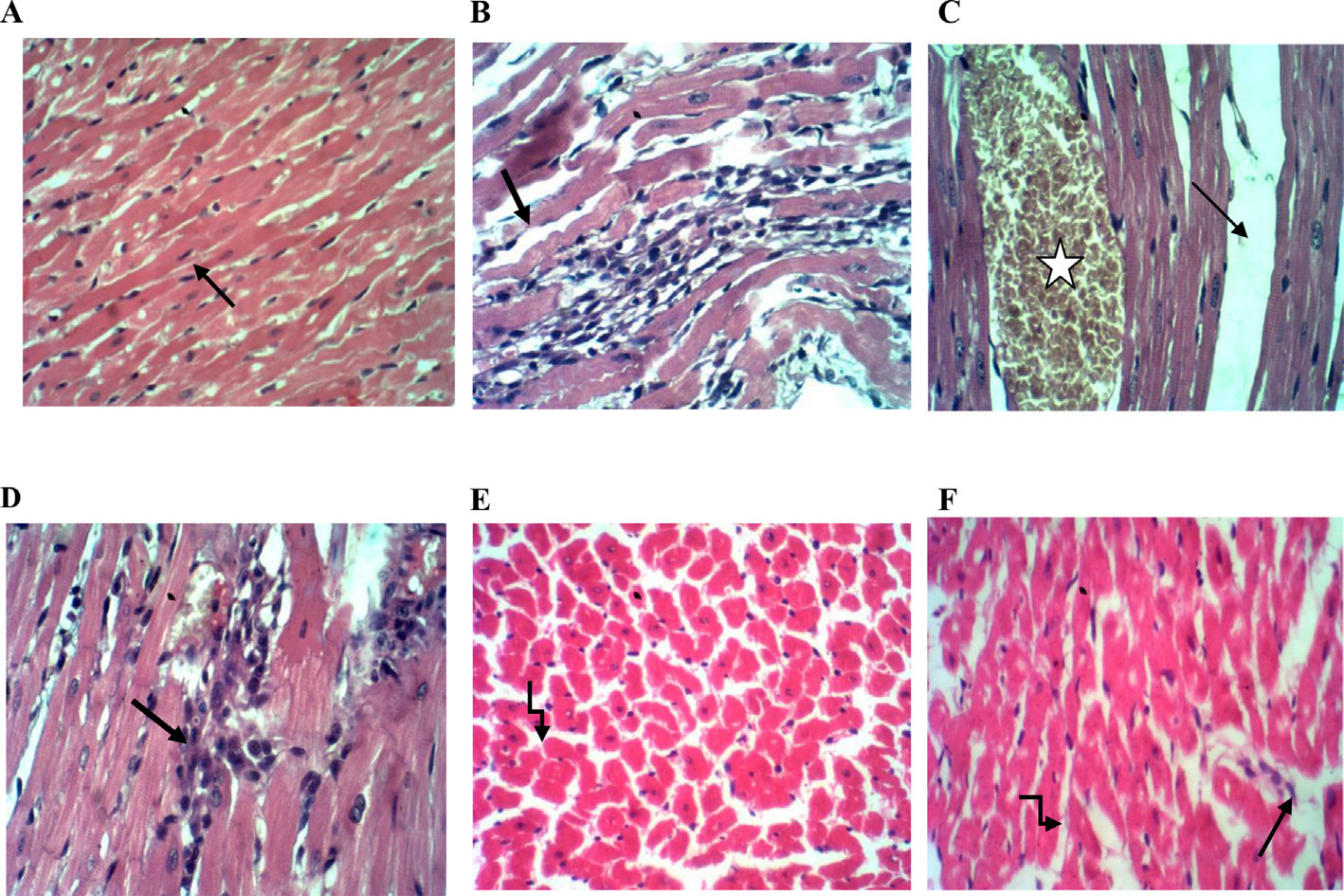
Representative light microscope photos for heart section stained with H&E of the three groups in the study. A: the normal control group showing normal cardiac myocytes with oval nuclei and regular cross striations B&C&D: ISP- treated group showing: B) wavy fibers (arrow) and interstitial inflammatory cell infiltrate, C) interstitial edema (arrow) and congested vascular space (star) D) focal inflammatory cell infiltration (arrow). E&F: ISP + TQ20 group showing cardiac myocytes with slightly separated myocardial fibers (broken arrow) and scattered few inflammatory cells (arrow) (H&E, x400).

**Figure 3:**
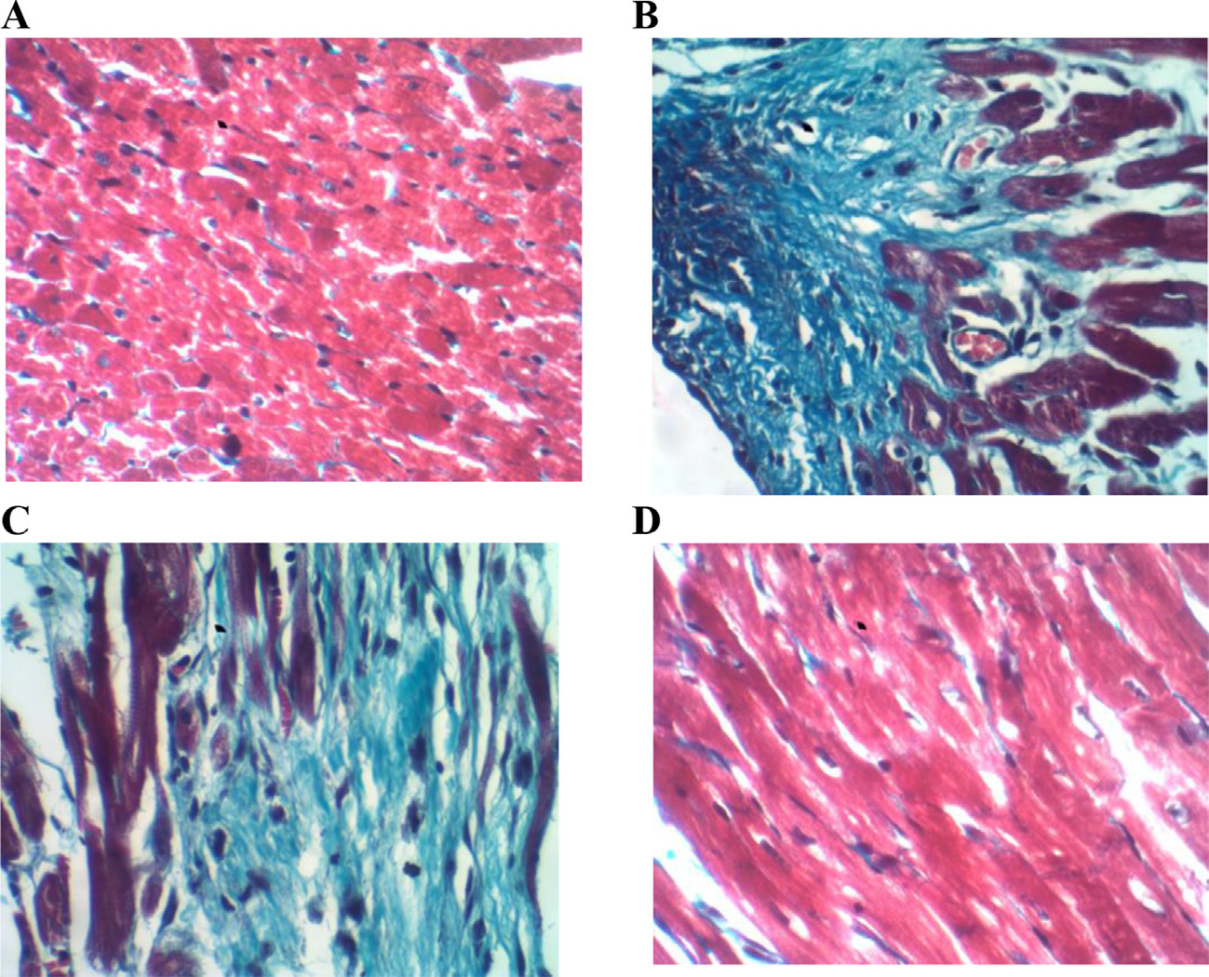
Representative light microscope photos for heart section stained with Masson's trichrome stain. A: the control group showing normal cardiac histoarchitecture with interstitial delicate collagen fibrils stained in blue. B&C: ISP group showing interstitial blue thick dense collagen fibrils associated with necrotic myocytes. D: ISP + TQ20 group showing few interstitial collagen fibrils. (Masson's trichrome stain, x400).

Heart sections stained with H&E from control rats demonstrated a normal histoarchitecture with the intact myocardial cell membrane, oval nuclei, and regular cross-striations, Figure 2A. In contrast, heart sections obtained from the ISP-treated group showed histological alterations in the form of myocardial cell separation caused by interstitial edema, wavy fibers, as well as inflammatory leukocyte infiltration, Figures 2 B&C&D. The pretreatment with TQ before the induction of MI in the (ISP + TQ20) group, showed some protective effects, including slightly separated myocardial fibers with few scattered inflammatory cells, as illustrated in figures 2 E&F. Heart tissue sections stained with Masson's trichrome taken from the control group demonstrated normal cardiac histoarchitecture with delicate collagen fibrils stained in blue, Figure 3A. On the other hand, the cardiac tissue sections from the ISP- treated group revealed pathological alterations in the form of interstitial thick dense collagen fibrils stained in blue, Figures 3 B&C. Interestingly, as shown in Figure 3D; the pre or co-treatment with TQ resulted in few interstitial collagen fibrils resembling the control group.

### 3.3 Effect of TQ pretreatment on oxidative stress markers

**Figure 4:**
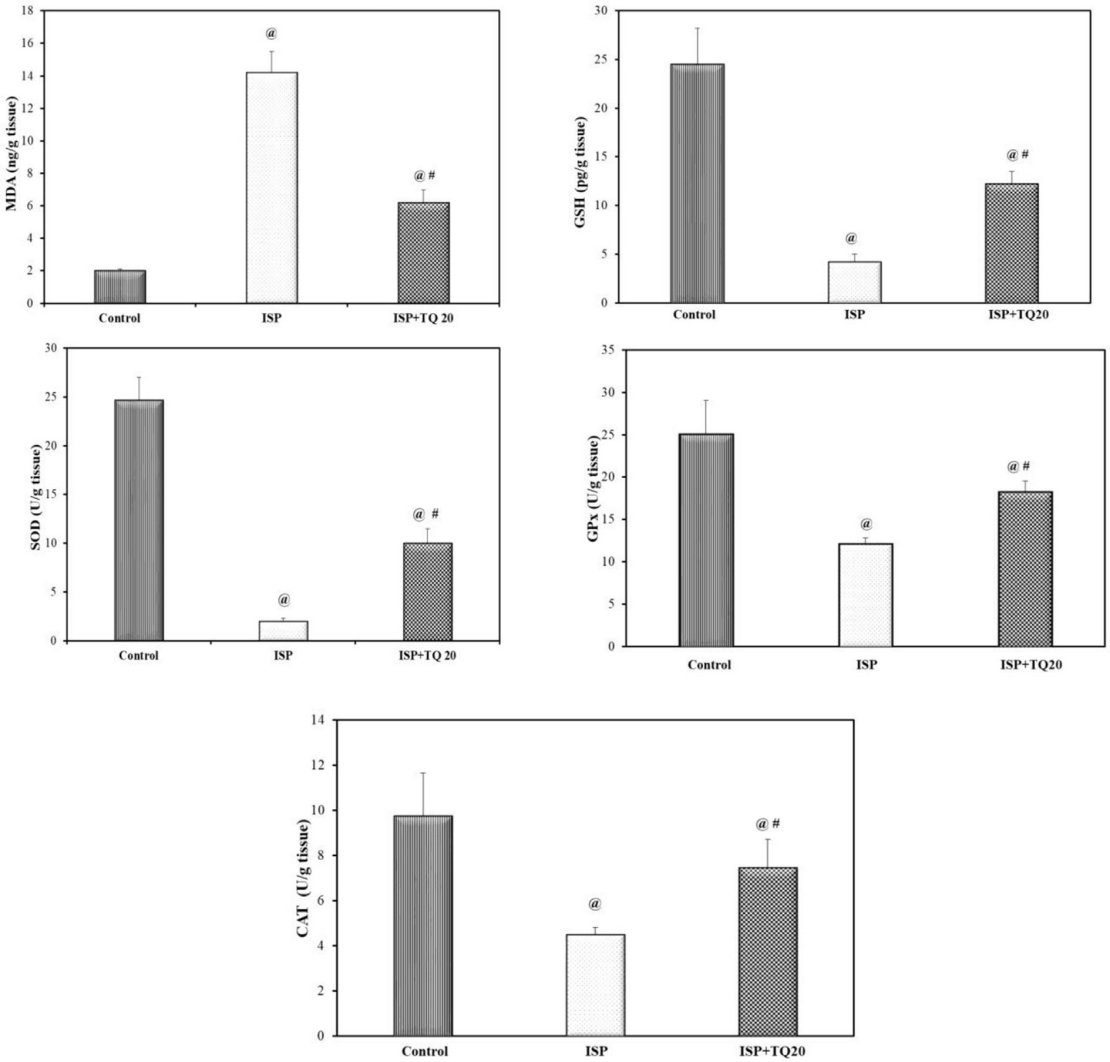
Effect of thymoquinone administration on cardiac tissue malondialdehyde (MDA) level, reduced glutathione (GSH) concentration, superoxide dismutase (SOD), glutathione peroxidase (GPx), and Catalase (CAT) activities. Values are Mean ± SEM, n = 6; @: compared with control, p < 0.05; #: with ISP group, p < 0.05.

Cardiac MDA, a lipid peroxidation marker, showed a significantly elevated level in the ISP-treated group compared to the normal control group. However, the pre-and co-treated group with TQ produced a significant decrease in MDA level as compared to the ISP-treated group (F = 361.67, p < 0.001). Reduced glutathione (GSH) was consumed in the ISP-treated group and its level was significantly reduced as compared to the control group, while GSH content was preserved and significantly elevated in the TQ pre and co-treated groups, compared to the ISP-treated group (F = 117.781, p < 0.001). The opposite occurred in the antioxidant enzymes; the cardiac SOD activity was significantly decreased in the ISP-treated group compared to its original level found in the normal control group. While the pre-and co-treated group with TQ showed a significant elevation in SOD activity as compared to the ISP-treated group (F = 384.17, p < 0.001), Figure 4. The activity of glutathione peroxidase was significantly decreased in the experimentally induced MI group as compared to the control one, while the pre-and co-treated group with TQ showed a significant elevation in the activity of glutathione peroxidase when compared to the ISP group (F = 42.416, p < 0.001), Figure 4. Moreover, catalase activity in the cardiac tissue was reduced significantly in the ISP-treated rats as compared to the control rats, while pre-and co-treatment with TQ showed a significant increase in the catalase activity as compared to the ISP-treated rats (F = 23.259, p < 0.001), Figure 4.

### 3.4 Effect of TQ pretreatment on pro-inflammatory markers

**Figure 5:**
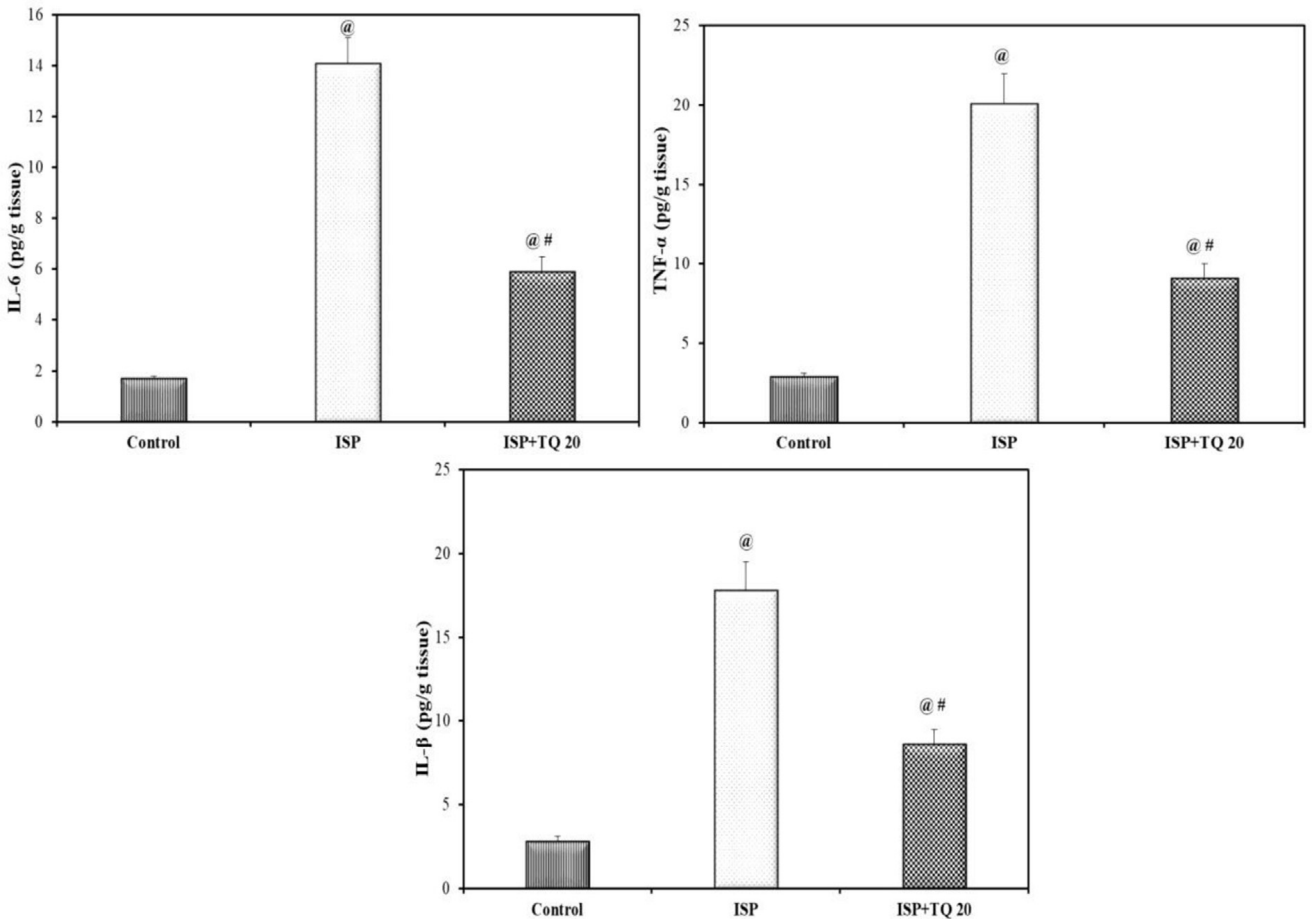
Effect of thymoquinone pretreatment on the level of cardiac tissue inflammatory markers: interleukin 6 (IL-6), tumor necrosis factor alpha (TNF-α), and interleukin 1β (IL-1β). Values are Mean ± SEM, n = 6; @: compared with control, p < 0.05; #: with ISP group, p < 0.05.

The pro-inflammatory cytokines associated with MI, including IL 6, TNF-α, and IL-1β, showed a significant increase in their tissue levels in the ISP-treated group when compared with the normal control group. Upon the pre-and co-treatment with TQ for 21 days, a significant decrease in the pro-inflammatory mediators’ tissue levels was detected as compared to the ISP-treated group (F = 702.22, F = 388.51and F = 350.14, respectively, p < 0.001) as shown in Figure 5.

### 3.5 Effect of TQ pretreatment on cardiac Bax, Bcl-2, and caspase-3 levels as apoptosis biomarkers

**Figure 6:**
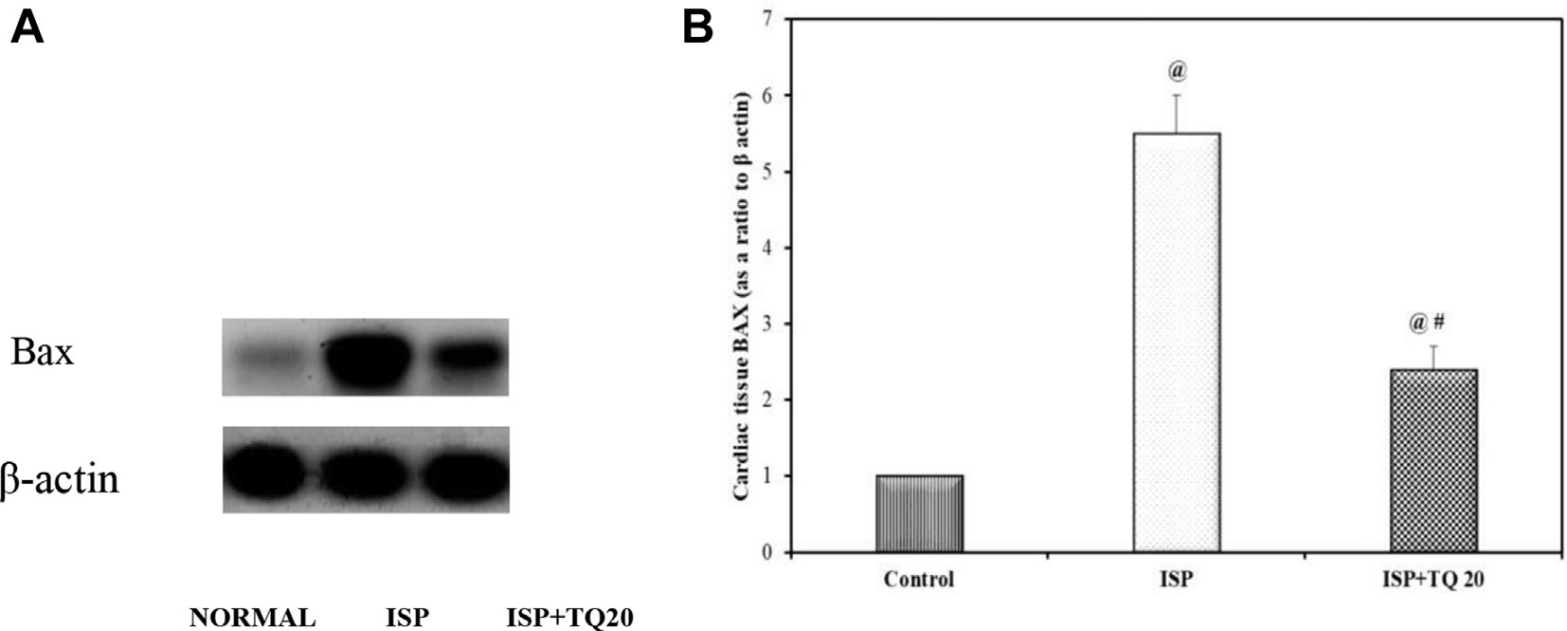
A): Immunoblotting for the Bax expression in cardiac tissue by the ChemiDocMP imager. (Supplementary material Fig. 6 A). B) Bar graph showing the impact of thymoquinone pretreatment on Bax level as a biomarker of apoptosis. Values are Mean ± SEM, n = 6; @: compared with control, p < 0.05; #: with ISP group, p < 0.05.

**Figure 7:**
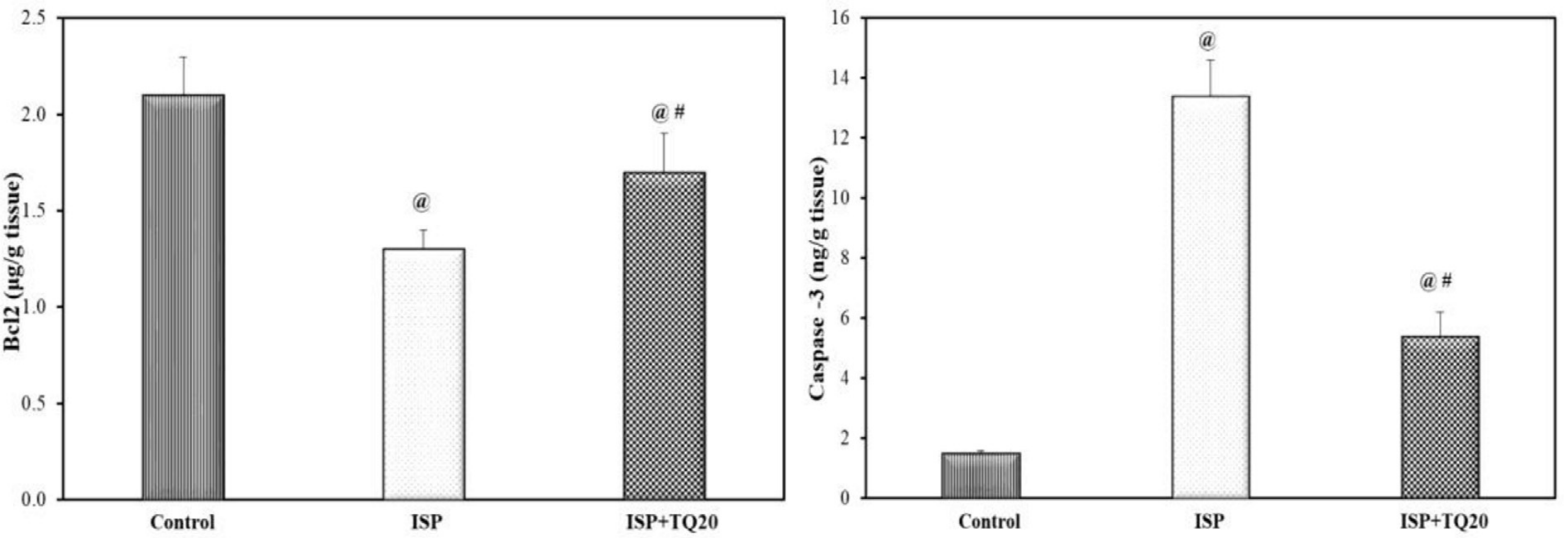
Effect of thymoquinone administration on the antiapoptosis marker Bcl2 level and on caspase-3 level as an apoptotic marker, in the cardiac tissue. Values are Mean ± SEM, n = 6; @: compared with control, p < 0.05; #: with ISP group, p < 0.05.

Bax protein, a reliable marker for intrinsic apoptosis, was detected by using the western blotting technique in cardiac tissues. The protein bands of Bax were quantified using the ChemiDocMP imager and normalized to β-actin protein bands and the results were expressed as a ratio between the content of the two proteins, Figure 6A. The level of Bax protein in the ISP-treated group increased significantly as compared to the normal control group. However, the pre-and co-treated TQ group produced a significant decrease in Bax protein content as compared to the ISP-treated rats (F = 267.34. p < 0.001), Figure 6B. The anti-apoptotic protein Bcl2 content demonstrated a significant decrease in the ISP-treated group as compared to the normal control group but showed a significant increase in the ISP + TQ20 group when compared to the ISP group (F = 29.035, p < 0.001), Figure 7. In contrast, administration of ISP alone produced a marked increase in the cardiac tissue caspase-3 level, a marker of cell apoptosis, as compared to the control group. The administration of TQ before and concomitantly with ISP showed a significant decrease in the caspase-3 level when compared to the ISP group. (F = 317.714, p < 0.001), Figure 7.

### 3.6 Effects of TQ pretreatment on cardiac mitochondrial (mtDNA)

**Figure 8:**
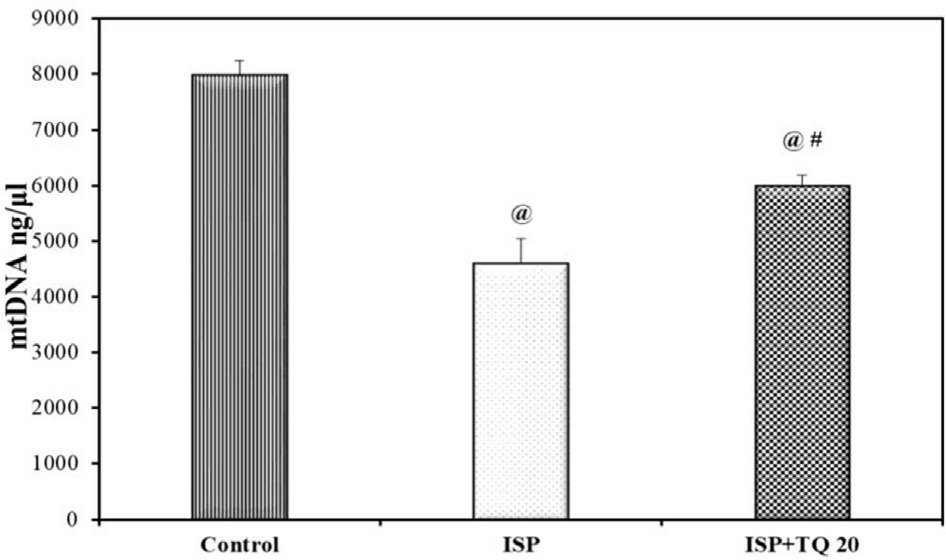
Effect of thymoquinone pretreatment on cardiac mitochondrial (mt DNA) content. Values are Mean ± SEM, n = 6; @: compared with control, p < 0.05; #: with ISP group, p < 0.05.

As presented in Figure 8, mitochondrial DNA (mtDNA) content was significantly decreased in the ISP-treated group of rats as compared to the normal control group. However, rats pretreated with TQ 20 mg/kg for 21 days showed a significant increase in mtDNA content when compared with the ISP-treated group, (F = 179.19, p < 0.001).

### 3.7 Correlations between cardiac mtDNA and serum cardiac markers, oxidative parameters, cardiac inflammatory mediators, and apoptosis markers in all experimental groups

As concluded in Table 1; correlations studies using all experimental groups (n = 18) showed significant associations (p < 0.001) between all identified markers in our research. A significant negative correlation was shown between both the serum cardiac marker LDH and c Tn I and cardiac mtDNA (r = -0.900 and -0.893, p < 0.001, respectively). Significant strong correlations were demonstrated between oxidative stress indices (MDA, GSH, SOD, GPx, and CAT) and mtDNA in the cardiac tissue. Also, a significant negative correlation was found between the cardiac mtDNA content and the lipid peroxidation marker; MDA (r = - 0.929, p < 0.001). In contrast, a significant positive correlation was found between cardiac mtDNA and the antioxidant markers (GSH, SOD, GPx, and CAT) (r = 0.946, 0.964, 0.807 and 0.912, respectively, p < 0.001). Moreover, a strong negative significant correlation was found between cardiac mtDNA and all pro-inflammatory cytokines (IL6, TNFα and IL-1β), (r = - 0.929, - 0.956, and - 0.957, respectively, p < 0.001). Similarly, a significant negative correlation was found between cardiac mtDNA and the apoptosis markers; Bax and caspase-3 (r = - 0.911,-0.932, respectively, p < 0.001), while mtDNA and the anti-apoptotic protein, Bcl2 demonstrated a strong significant positive correlation between them (r = 0.849, p < 0.001).

**Table 1:** Correlations between cardiac mtDNA and oxidative stress markers (malondialdehyde; MDA, reduced glutathione; GSH, superoxide dismutase; SOD, glutathione peroxidase; GPx, catalase; CAT), inflammatory markers (interleukin 6; IL6, tumor necrosis factor alpha; TNF-α, and interleukin 1β; IL-1β) and apoptosis markers (Bax, Bcl2, and caspase-3). Correlation coefficient using person's test was made between the rats in all experimental groups.

	Markers	Correlation coefficient (r)	
Cardiac tissue mtDNA	Cardiac Oxidative stress markers	MDA	-0.929∗
		GSH	0.946∗
		SOD	0.964∗
		GPx	0.807∗
		CAT	0.912∗
	Cardiac Inflammatory markers	IL-6	- 0.929∗
		TNFα	- 0.956∗
		IL-1β	- 0.957∗
	Cardiac Apoptosis marker	Bax	- 0.911∗
		Bcl2	0.849∗
		Caspase-3	-0.923∗

∗Correlation is significant at p < 0.001, n = 18.

## 4 Discussion

Thymoquinone is a natural component that has antioxidant and anti-inflammatory properties, rendering it an effective choice as a cardioprotective agent against ischemic heart diseases, including MI. However, the detailed cardioprotection mechanism of TQ requires further investigation [22, 27]. Our study was designed to investigate more about the mechanism of action of TQ in ISP induced MI in rats and explore its effect on oxidative stress markers, inflammatory cytokines, apoptotic markers, and the cardiac content of mtDNA.

The results of our study demonstrated that the administration of TQ for 21 days before and at the same time of MI induction showed a significant decline in serum cardiac biomarkers and cardiac lipid peroxidation levels, together with significant improvement in cardiac antioxidant activities and levels. In addition, pre- and co-treatment with TQ resulted in a significant decrease in the levels of inflammatory cytokines IL6, TNF-, and IL-1β, as well as a significant protection against cell death. Furthermore, the cardioprotection of TQ extends to preserving the cardiac mtDNA content, which in turn keeps energy production in the cardiomyocytes and helps the cardiac tissue to cope with its dynamic nature.

The induction of MI in our study was done by subcutaneous injection of isoproterenol (ISP), which is a synthetic catecholamine with β-adrenergic agonist activity that leads to a positive chronotropic and inotropic effect, which in turn causes severe cardiac stimulation and imbalance between the high cardiac demand and decreased coronary blood flow [43]. Moreover, ISP produces highly cytotoxic-free radicals and ROS, which leads to another imbalance between the free radicals and antioxidative defense, resembling the MI that occurs in humans [27].

Serum cardiac biomarkers LDH and cTnI are reliable indicators of the incidence of MI [44, 45]. Furthermore, cardiac Troponin cTnI is considered as the gold standard biomarker used in the diagnosis of myocardial infarction [46]. In the present work, the induction of MI was confirmed by the significant elevation of serum LDH and cTnI levels in the ISP-treated rats as compared to the control group. However, the TQ pre-and co-treated group demonstrated a decline in these serum biomarker levels. This finding reveals the cardioprotective effect of TQ against cardiac injury. The decrease in the level of serum cardiac biomarkers upon the administration of TQ was matched with previous studies conducted by Lu, Feng [21], and Hassan, Akhtar [22].

Moreover, the histopathological findings from the myocardium of ISP-treated rats showed interstitial edema, congestion, and inflammatory cell infiltration. Unlike normal control rats that demonstrated normal cardiac histoarchitecture. While the pre-and co-treatment with TQ was able to prevent the injurious alterations made by ISP injection. Thus, these findings confirmed the establishment of MI by ISP and the impact of TQ use in the alleviation of the morphological damage associated with MI. Our H&E histopathological findings were confirmed by Asgharzadeh, Bargi [47] and, Randhawa, Alghamdi [48].

Masson's trichrome staining was also used to illustrate fibrotic changes and collagen deposition associated with myocardial infarction injury [45]. In the present work, heart tissue sections from the ISP group showed dense and thick blue collagen fibrils as compared to the control group, which showed only a few delicate collagen fibrils. This confirms the induction of myocardial infarction using ISP. However, pre-and co-treatment with TQ resulted in heart tissue sections closely similar to those obtained from the control group, which indicates the cardioprotective effect of thymoquinone in the form of decreasing fibrous tissue formation. Previous studies have reported the antifibrotic effect of thymoquinone on the liver [49], kidney [50], and heart tissues [47], which all support our work.

Myocardial infarction (MI) is associated with excessive production of ROS, depletion of antioxidant defenses, and incidence of oxidative stress state, which is responsible for a cascade of pathological events that induce both functional and structural damage of cardiomyocytes. In the present study, the incidence of oxidative stress in the ISP-treated group was demonstrated by multiple changes in oxidative stress biomarkers, including the significant elevation of the level of MDA, the end product of lipid peroxidation, the significant inhibition of the nonenzymatic antioxidant biomarker GSH and the enzymatic antioxidant biomarkers including SOD activity, glutathione peroxidase, and catalase activity. However, pre-and co-administration of TQ for 21 days before the induction of MI significantly attenuated the oxidative stress, which indicates the antioxidant properties of TQ. These findings indicate the ability of TQ to fight against the oxidative stress state associated with the incidence of MI. This state is responsible for numerous injuries in cardiac tissue, ranging from cell membrane injuries to subcellular injuries. The powerful antioxidant effect of TQ was supported by previous studies conducted by Ojha, Azimullah [27], Lu, Feng [21], and Pei, Hu [51].

Cardiomyocytes contain a high number of mitochondria, which act mainly as a source of energy and an oxidative stress regulator [52]. Mitochondria are the main site of ROS production, and at the same time, they are extremely affected by the excessive amounts of ROS produced during uncontrollable oxidative stress states such as that occurred with MI [53]. The most sensitive part of the mitochondria is its unique circular mtDNA, which is highly susceptible to oxidative damage by excessive ROS. Possibly because of its proximity to the respiratory chain in the mitochondrial inner membrane or the lack of protective histone-like proteins and its poor repair activity against damage. So, excessive mitochondrial ROS production may result in severe destruction of the mtDNA and loss of mitochondrial functions [13].

In the present work, mtDNA content in the cardiac tissue was significantly decreased in the ISP-treated group, while significantly preserved in the (ISP + TQ20) group. These results revealed the protective impact of TQ on the cardiac content of mtDNA. Khan, Vaibhav [54], Silachev, Plotnikov [55], and Abdelmeguid, Fakhoury [56] have reported the protective effects of TQ on mitochondria in β-amyloid-induced neurotoxicity, brain ischemia/reperfusion injury and in streptozotocin-induced diabetes respectively. However, the exact mechanism regarding the restoration of mitochondria dysfunction and the molecular effect of TQ on mtDNA is not fully elucidated.

In our study, a significant negative correlation between MDA level and mtDNA content in the cardiac tissue, together with a significant positive correlation between the internal cardiac antioxidants (GSH, SOD, GPx, and CAT) and cardiac mtDNA content was found. These correlations can explain the possible role of increased MDA release and depletion of GSH, content, and SOD, GPx, CAT activities during oxidative stress injury in the damage of mtDNA. The correlation between mtDNA content and oxidative stress state was reported by Ilya V. Chestkov, Jestkova [57], and Ide, Tsutsui [14].

To our knowledge, the present study may be considered the first study to give attention to the protective effect of TQ on cardiac mtDNA in experimentally-induced MI in rats. The plausible explanation of preservation of cardiac mtDNA by TQ may contribute to the antioxidant properties of TQ, which prevents the damaging effect of ROS on the cardiac cells.

The present work gives a focus on the changes that occurred in the inflammatory cytokines. Many studies have reported that MI is associated with inflammation and the release of pro-inflammatory cytokines, including IL6, IL1β, and TNFα [58, 59, 60, 61]. Results from the present study showed that the proinflammatory cytokines were significantly elevated in ISP-induced MI in rats, while the (ISP + TQ20) group showed a significant decrease in the level of the pro-inflammatory cytokines. The anti-inflammatory properties of TQ have been documented by many studies conducted on the cardiac tissue [27], central nervous system [62], and rheumatoid arthritis [63].

Nakayama and Otsu [64] demonstrated that with the incidence of oxidative stress or exaggerated inflammation, such as in MI, the mtDNA becomes fragile and easily degraded. Also, when mtDNA fragments are released to the intracellular cytosol, they trigger cardiac inflammation and stimulate the signaling of pro-inflammatory cytokines including TNF-α, ILβ, and IL-6 [65], which exaggerate the inflammatory process, followed by more damage of mtDNA and loss of the mitochondrial role [66].

A negative correlation demonstrated in the present study between the cardiac pro-inflammatory cytokines and cardiac mtDNA content, may explain the role of the increased pro-inflammatory mediators in damaging mtDNA, decreasing its content in the cardiac tissue. mtDNA damage has been observed in chronic neuroinflammatory disorders [67]. The pre-and co-administration with TQ has decreased the pro-inflammatory cytokines and has protected the mtDNA damage, increasing its cardiac content via its potent anti-inflammatory impact.

Cardiomyocyte cell death in the peri-infarct zone is largely due to apoptosis, which is accompanied by many morphological and functional alterations [17, 68]. As reported by many studies, Bax (pro-apoptotic protein) is activated by cellular oxidative stress as in MI, which triggers the intrinsic mitochondrial pathway of apoptosis [69, 70]. This strongly correlates with our finding, where Bax protein was highly increased in the ISP-treated group due to the increased oxidative stress and inflammation. In addition, our results revealed that upon the pre-and co-administration of TQ, the amount of Bax protein was significantly declined. This was supported by many studies that reported the use of TQ in different ischemic models [71, 72], inhibiting the pro-apoptotic Bax protein. As documented by D. Westphal et al. [73], during the apoptosis process, both Bax and Bak oligomerize to form pores called mitochondrial outer membrane permeabilization (MOMP) in the mitochondrial outer membrane that enables the release of mtDNA in cell death [68, 74]. This sequential information supports our finding regarding the significant negative correlation between the cardiac mtDNA content and Bax level. While the pre-and co-treatment with TQ inhibited the mitochondrial-induced apoptosis and decreased the Bax protein level and hence protected the mtDNA.

The anti-apoptotic protein Bcl2 plays an important role in cardioprotection during ischemic situations [75]. In the present study, induction of MI using ISP showed a significant decrease in the level of Bcl2 as compared to the control group, while the use of TQ demonstrated an anti-apoptotic effect through a significant increase in the Bcl2 level as compared to the ISP-treated group, a similar finding was reported by Lu et al [21]. A strong positive correlation was found between the Bcl2 level and cardiac mtDNA content. This finding was supported by a previous study carried out by lindasy et al. [76], which reported that Bcl-2 proteins are localized on the outer mitochondrial membrane and inhibit the activation of the pro-apoptotic family members Bax and Bak, thus inhibiting apoptosis and mtDNA release. So, the increased level of Bcl2 via pre-and co-treatment of TQ has a role in the preservation of cardiac mtDNA content.

The caspase-3, the reliable marker of apoptosis and the final common product of the extrinsic and intrinsic apoptotic pathways, was significantly increased in the ISP group [77]. The persistent apoptosis after MI leads to myocardial fibrosis, cardiac remodeling, and pathological alteration in cardiac function [78, 79]. The results of our study revealed that pre-and co-treatment with TQ in the third group significantly attenuated apoptosis by decreasing the myocardial tissue level of caspase-3, which indicates the anti-apoptotic properties of TQ. A significant strong negative correlation was also found between caspase-3 level and cardiac mtDNA content, which indicates that the pre-and co-treatment with TQ prevents caspase-3 activation, thus inhibiting apoptosis and preserving the cardiac mtDNA content. These findings are in agreement with a previous study conducted by Xiao et al., and Galaly et al. [72, 80].

## 5 Conclusion

In conclusion, TQ is a natural product with antioxidant, anti-inflammatory, and anti-apoptotic properties. In addition, it protects against the damage of the cardiac mtDNA associated with MI, which in turn keeps the energy production in the dynamic cardiomyocytes and adds more value to the cardiac benefits obtained from the use of TQ as a natural cardioprotective agent.

## Declarations

### Author contribution statement

Asmaa Khalifa: Performed the experiments; Analyzed and interpreted the data; Wrote the paper.

Radwa Rashad: Analyzed and interpreted the data; Wrote the paper.

Wessam El-Hadidy: Conceived and designed the experiments; Wrote the paper.

### Funding statement

This research did not receive any specific grant from funding agencies in the public, commercial, or not-for-profit sectors.

### Data availability statement

Data included in article/supplementary material/referenced in article.

### Declaration of interests statement

The authors declare no conflict of interest.

### Additional information

No additional information is available for this paper.
