# Supplementary material for: Thymoquinone protects against cardiac mitochondrial DNA loss, oxidative stress, inflammation and apoptosis in isoproterenol-induced myocardial infarction in rats
Source: Heliyon. 2021 Jul 14;7(7):e07561. doi: 10.1016/j.heliyon.2021.e07561 (PMC8322274; doi:10.1016/j.heliyon.2021.e07561)

**Supplementary material of Figure 6 A):** Immunoblotting for the Bax expression in cardiac tissue by the ChemiDocMP imager.

Bands of Bax


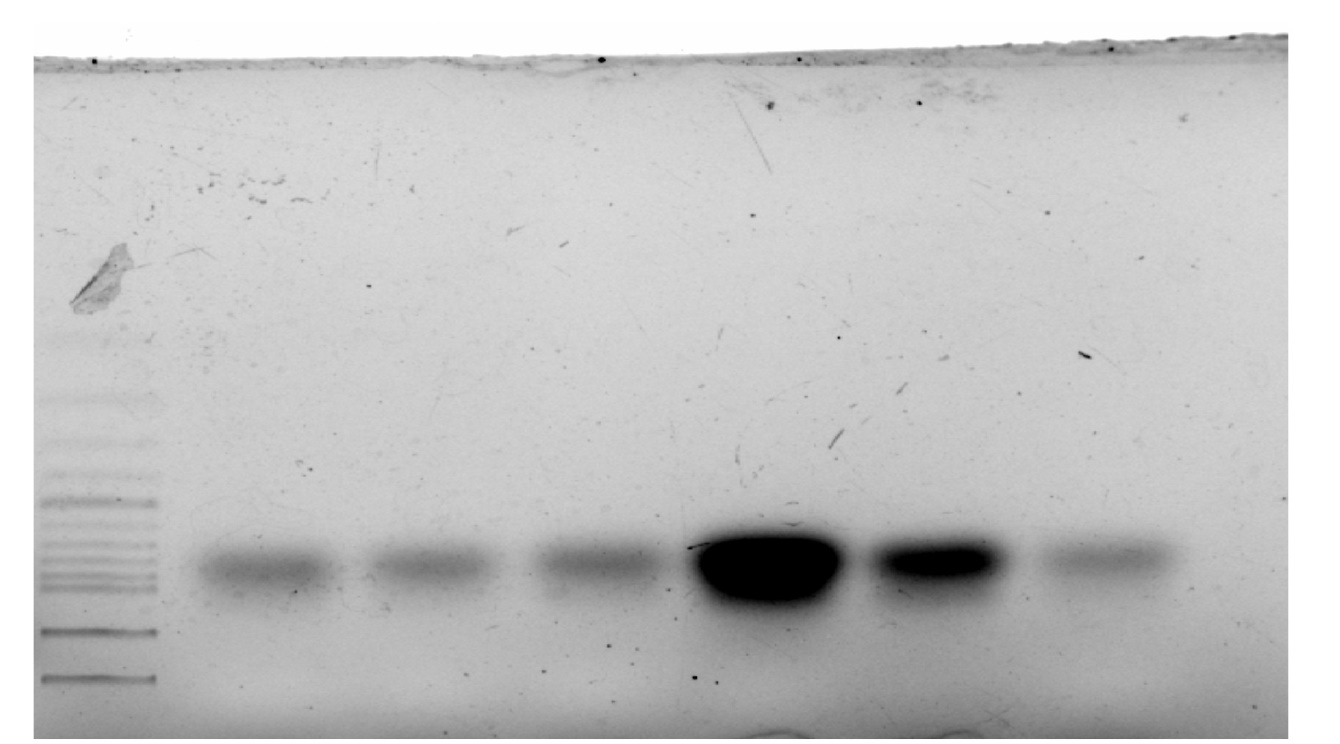


bands of Β –actin:


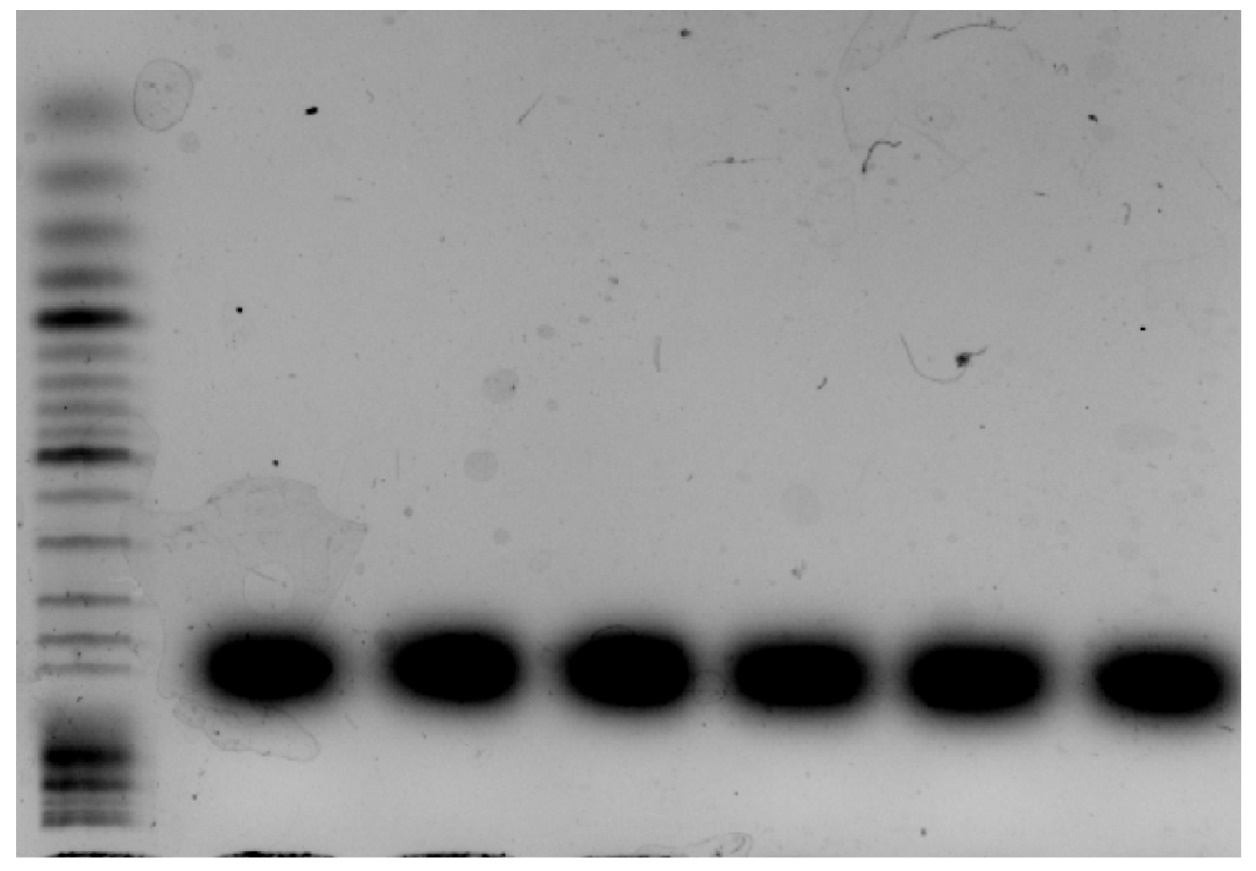

Supplement: Supplementary material Fig. 6 A [file mmc1.docx]
